# Supplementary material for: Search for a genetic cause of variably protease-sensitive prionopathy
Source: PLoS Pathog. 2025 Aug 1;21(8):e1013343. doi: 10.1371/journal.ppat.1013343 (PMC12327619; doi:10.1371/journal.ppat.1013343)
Supplement: Attachment — Submitted filename: R2R_combined_round1_and_round2.pdf [file ppat.1013343.s004.pdf]

# Response to Reviewers

*Search for a genetic cause of variably protease-sensitive prionopathy*
*Lian et al 2025*

**<u>Reviewer #1</u>**

**Summary:**
**In their submission to PLOS Genetics, Yian et al. present a detailed analysis of genetic information from a cohort of 67 variably protease-sensitive prionopathy patients (VPSPr). This article addresses a gap in knowledge wherein it remains uncertain if there is a genetic cause or the potential for familial inheritance of VPSPr.**
**Their analysis included exome sequencing and targeted sequencing of PRNP non-coding regions on genomic DNA from VPSPr patients in their cohort. The results of extensive genetic characterization of the patients in this cohort did not reveal genetic variants that are likely to cause VPSPr. The analysis of exome sequencing results in seqr filtering allele frequencies via two methods (filtering allele frequency in gnomAD and the allele frequency among 18,371 internal controls), and targeted sequencing of the genomic region including PRNP comparing allele counts. These results repeatedly suggested that the known M129V polymorphism in PRNP increases risk for VPSPr which has been reported previously and acts as a positive control for genetic risk in this study.**
**Overall, this work represents a thorough genetic approach to attempt identification of potential causal genetic factors in VPSPr patients. The use of a verified patient cohort in this study is a strength. The limitations of the study (lack of whole genome sequencing, and lack of matched controls) are acknowledged by the authors. The authors also acknowledge the difficulty of proving a negative hypothesis, but do succeed in putting forth a novel genetic analysis of VPSPr patients which supports their negative hypothesis – that VPSPr may be a sporadic condition with no heritable genetic cause.**

We thank the reviewer for this critical read of our work.

**Minor Points:**
**1) On page 14, a sentence reads "A handful of variants exhibited OR as high as 1.3 or as low as 0.8 in the sCJD GWAS without showing any signal in VPSPr, but the converse — variants associated with VPSPr but with sCJD — was not identified (Figure 4B), again suggesting no independent risk variants". This sentence seems incomplete. Should this say "the converse – variants associated with VPSPr but not with sCJD – was not identified…"?**

Correct, we thank the reviewer for noticing this error. The sentence has been fixed to read "variants associated with VPSPr but <u>not</u> with sCJD"

**2) On page 10, Yian et al. describe their analysis of 25 neurodegerative risk genes in their 67-patient cohort, and report only 2 instances of patients carrying Parkinson disease risk alleles. What 25 neurodegeneration risk genes were used for this analysis? This is of interest as there are currently more than 100 known genetic risk factors for Alzheimer's disease alone (and at least 24 for Parkinson disease). This explanation of familial history of dementia is a weak point in the article and could be expanded on. For example, if polygenic risk scores for dementias are possible or if an analysis of more neurodegeneration risk genes could be done, it would greatly increase the understanding of how familial risk of dementia may influence VPSPr or prionopathy more generally.**

The reason our list was only 25 is that it was limited to Mendelian genes, not GWAS hit genes. Accordingly we added the following to Methods: "We used a previously curated list[32] of 25 Mendelian disease genes that are associated with Alzheimer's, Parkinson's, frontotemporal dementia/amyotrophic lateral sclerosis, and Huntington's disease, ultimately sourced from Online Mendelian Inheritance in Man (OMIM) https://omim.org Because our question was whether *familial* forms of these neurodegenerative disorders are associated with risk of VPSPr, we did not include genes associated with these disorders through GWAS."

At the reviewers suggestion, we also looked into whether analysis of polygenic risk scores could be an interesting addition to this study. Unfortunately, all of the polygenic risk scores available for AD and PD rely on genome-wide SNP data, which we do not have for our cohort. For VPSPr, we have exomes, but there are currently no data validating how much the subset of PRS coefficients for variants found in the exome actually have predictive value independent of the rest of the genome. We also have targeted sequencing around *PRNP* but this region is not currently known to contribute significant risk to commoner dementias. Therefore we concluded that PRS is not a valid strategy given the data available in this study.

**<u>Reviewer #2</u>**

**In this manuscript, Lian et al. describe their effort to identify potential genetic causes underlying VPSPr by sequencing non-coding regions of the PRNP gene and the exome of 67 autopsy-confirmed patients. No causal variants for VPSPs could be identified. Prnp is of interest because it is an essential susceptibility gene for CJD and scrapie infections.**

We thank the reviewer for their critical read of our work.

**VPSPr is a rare "variably protease sensitive" PrP disease described by the Gambetti group (National Prion Disease Pathology Surveillance Center) as a "new sporadic disease of the prion protein". That group defined this human Creutzfeldt-Jakob Disease (CJD) subset in 5 cited papers, and additional cases were processed by the Prion Center for evaluating human brain histopathology and vole transmission studies. Only representative PrP Western blots for each 129 genotype (MM, VV, MV) were shown.**

**While a high proportion of VPSPr "patients appear to have a family history of dementia", none of these family members were diagnosed with sporadic CJD. The variable findings in the VPSPr patients, and especially in the vole transmissions, showed inconsistent lesion profiles, including unusual white matter and cerebellar disease. This data strongly suggest these patients comprise a heterogeneous group. Without more primary data on selected patients with consistent patterns of disease, infectivity, and PrP protease sensitivity, the DNA findings here should be published as a short, specialized communication.**

We have added Western blots per the reviewer's request, see point #1 below.

**The lack of variant alleles in DNA extracted from brain and blood was confined to a 152kb Prnp region. The authors might consider the following in a revision. The 152kb region is only 6% of the 26Mb DNA of the short arm of chromosome 20 where Prnp resides. Other factors and genes can affect susceptibility and disease progression, and this should be clarified for readers.**

We have added to the limitations paragraph: "Even near *PRNP*, our targeted sequencing does not capture all distal variation that might potentially be associated with expression of this gene."

**OTHER:**
**1) The current study does not show any data on their cases (specimen) that confirms unusual PrPCJD digestion by Western blot.**

We have added a supplementary file with Western blots of 10 VPSPr cases alongside negative and positive (sCJD) controls.

**2) It is not clear, from the methods or data shown, if individual cases by DNA analysis were distinguishable by their MM, MV or VV 129 PrP genotypes. The authors also ignore the isoleucine/met variants that were treated separately in the vole transmission paper. Which of their DNA analyses were verified as infectious cases and were they distinct from non-transmissible cases?**

Yes, the codon 129 genotypes were indeed determined by DNA sequencing. We have edited for clarity in the Results: "All 3 *PRNP* codon 129 genotypes were represented in our cohort".

The codon 109 isoleucine/methionine variant is found only in bank vole; all humans sequenced to date have had methionine at this codon.

This manuscript does not report any data on transmission of VPSPr to bank voles or other animals, so discrimination of infectious vs. non-transmissible cases is out of scope for this paper.

**3) Sporadic CJD cases (n=14 patients) have been reproducibly transmitted to hamsters with a 350-450 day primary passage incubation time that was reduced to <200 days on secondary passage. In contrast, 32 of the 141 VPSPr cases were transmissible to voles on first passage and some of these showed an incubation time of >800 days with 1 of 20 positive takes; moreover, only 6/9 of 1st passage takes could be confirmed on secondary vole passage. For a full paper, hamster transmissions (that are also susceptible to scrapie as well as sCJD) would be more informative for concluding that all VPSPr cases were infected with sporadic CJD.**

We agree that the existing literature data on transmissibility of VPSPr is rather incomplete and has not examined a large number of cases nor a variety of possible acceptor species. Transmission experiments are unfortunately out of scope for this paper on genetic sequencing to identify VPSPr risk factors.

**4) The introduction is very speculative ("hypothetical") and introduces a risk factor that seems convoluted and hard to follow with respect to other epidemiological data on actual cases. It would help if the generally accepted incidence of 1.2 per million of human prion diseases is used as a reference. Inverting this number and then multiplying the risk by the number of years one is alive seems questionable. The Prion Center also does not get all cases since CJD is not a reportable disease. The Prion Center also reported that VPSPr represents only 2.2% of all prion cases, i.e., 1.8 per hundred million, indicating a very low risk.**

We thank the reviewer for this comment. In response to this comment as well as reviewer #3's critique, we have downplayed the discussion of the family history for VPSPr.

<u>**Reviewer #3:**</u>

**This is a clear paper of high quality that should be published. I am confident their conclusions are correct.**

We thank the reviewer for this critical read of our paper and for the helpful suggestions below.

**Some suggestions:**
**I am not so convinced by the family history argument, the authors cite a review paper which quotes a family history of dementia (not detailed how this was defined) in 15/36 cases (42%). There was not control group here. In studies and in biobanks a reported family history of dementia is in the range 10-20% the lifetime risk of dementia is in keeping with this approx range too. I suspect the rate seen in VPSPr might be statistically significantly increased in a formal study, but this has not been done. My read of the current text is that the authors assume for the purposes of calculations a family history of (undiagnosed) VPSPr in 42%. This is surely far from correct, in fact my default position would be that none of the 42% postive family history is explained by VPSPr (as is the case in sCJD which also has an increased rate of family history of dementia I think Zerr**

**was senior author on that paper from about 15 yrs ago). I dont think there is a report of 2 occurrences of VPSPr in any one family. Therefore I find the time spent on this analysis out of keeping with the quality of the information and the paper would be better with this simply removed. I think its perfectly fine to look for explanatory mutations using exome sequencing, it doesnt need this justification which I think is just misleading for people.**

We thank the reviewer for this suggestion. Particularly in light of the negative results reported here, we agree it is appropriate to reduce the amount of discussion of the supposedly elevated rate of family history in VPSPr. Accordingly we have deleted all references to VPSPr family history from the Abstract and the Author Summary. In the Introduction, we left in a reference to it but with an added caveat referencing Krasnianski 2012, which appears to be the Inga Zerr paper the reviewer referred to:

"Although controlled studies have not been done to determine whether this rate of family history of dementia differs from other diagnoses, it is nominally higher than the 12.1% reported in sporadic CJD [Krasnianski 2012 PMID 17822808], leading to speculation that VPSPr might be a form of genetic prion disease with a cause other than PRNP coding variants."

In the Results, we decided we could not fully delete the discussion of family history, because without it, it would be impossible to explain our genetic search strategy. In particular, if one had no prior reason to believe VPSPr might be caused by a modestly penetrant Mendelian variant, then one would not be likely to choose to perform targeted sequencing and exome sequencing without a large number of matched controls. This study design choice was justified by the hypothesized genetic architecture based on these reports of elevated family history. Absent that hypothesis, depending on one's assumptions, one might have chosen a GWAS design or an exome burden test design with matched controls. Therefore we have left in some reference to the family history in the Results and Discussion, but have added in caveats for example:

"While many genetic architectures for VPSPr are possible, as a starting point, we considered the possibility that the 42% rate of positive family history is due to a modestly penetrant single genetic variant."

"VPSPr, if its 42% positive family history were indeed explained by a genetic cause, could plausibly arise from variants with OR > 1,000"

"Well-controlled studies have never examined the frequency of positive family history of dementia in VPSPr versus sporadic CJD or other rapidly progressive dementias. Nonetheless, the positive family history reported in 42% of VPSPr cases has led several investigators to speculate whether VPSPr has a genetic cause."

We also referenced the Inga Zerr paper again in the Discussion:

"CJD was also reported to have a higher rate of family history than found in matched controls [Krasnianski 2012 PMID: 17822808], even though GWAS has since shown that sCJD has relatively low heritability (h2 = 0.24 to 0.26)[34]."

We hope that these changes strike the right balance of justifying the study design choices while not overcrediting the prior claims of elevated family history, which as the reviewer points out, were never supported by extremely strong evidence.

**The authors conclude the disease should be grouped with sCJD as a sporadic disease. I agree. I would be interested therefore if risk factors reported in the recent sCJD GWAS are enriched in VPSPr, the STX6 and GAL3ST1 variants linked to sCJD are in the ORF or have perfect proxies in the trancripts so should be covered by the exome sequencing. I appreciate there are no sequenced controls, but there are publicly available data.**

We have added a Table 3 comparing the frequencies of these tagging SNPs in VPSPr, sCJD, and gnomAD. There were no obviously significant differences.

**Have all data underlying the figures and results presented in the manuscript been provided?**
**Large-scale datasets should be made available via a public repository as described in the PLOS Genetics data availability policy, and numerical data that underlies graphs or summary statistics should be provided in spreadsheet form as supporting information.**
**Reviewer #1: Yes**

**Reviewer #2: No: per review no data provided for protease resistance of PrP of brain specimen analyzed (needed of at least 10 different samples with sCJD controls) or relevant infectivity studies of their cases (as a table of their cases from the Prion center).**

We have added a supplementary file with Western blots of 10 VPSPr cases alongside negative and positive (sCJD) controls.

**Reviewer #3: No: Data are deposited in dbGAP, with controls around consent and the intent of the research. It is not clear from the paper alone whether these data will be possible to access.**

dbGaP deposition is in progress. As of this writing, the Institutional Certification and Basic Study Information have been provided to the individual at NINDS responsible for dbGaP deposition, and we are awaiting further instructions. Note that all summary statistics are provided in the study's GitHub repository and are sufficient to reproduce the analyses herein.

**PLOS authors have the option to publish the peer review history of their article (what does this mean?). If published, this will include your full peer review and any attached files.**

**If you choose “no”, your identity will remain anonymous but your review may still be made public.**
**Do you want your identity to be public for this peer review? For information about this choice, including consent withdrawal, please see our Privacy Policy.**
**Reviewer #1: No**
**Reviewer #2: Yes: Laura Manuelidis MD, Professor, Yale Medical School**
**Reviewer #3: No**

We agree to the publication of the peer review history of this article.

# Response to Reviewers Round 2

*Search for a genetic cause of variably protease-sensitive prionopathy*
*Lian et al 2025*

**Reviewer #1: In their submission to PLOS Genetics, Yian et al. present a detailed analysis of genetic information from a cohort of 67 variably protease-sensitive prionopathy patients (VPSPr). The authors addressed reviewer comments in this resubmission. Although the scope of added data was limited, the addition of Table 3 adds some value. The authors have tempered their claims, and addressed the study's limitations more clearly which is appreciated. This manuscript provides data to support the authors hypothesis that VPSPr is a sporadic disease including valuable genetic data and from VPSPr clinical cases.**

**Minor Points:**

**1) The authors repeatedly mention the presence of tau, amyloid-beta, or synuclein pathology in diagnosed VPSPr cases. Can the authors provide any information about the co-occurrence of these other pathologies in the cohort in this study? The interest here is two-fold. Firstly, this information could be helpful in assessing the "family history" that the authors discuss - as there is known genetic risk within the presumed etiologies of tau, amyloid-beta, and alpha-synuclein pathology. Secondly, does susceptibility to tau, amyloid-beta, alpha-synuclein, and prion pathologies somehow interplay? This question goes beyond the scope of this manuscript, but the presence of other pathologies was presumably recorded in these patients, and can at least support the idea that these pathologies do coincide with prions in VPSPr.**

These cases were diagnosed through the standard U.S. prion surveillance autopsy program; assessment of co-pathologies is not routinely performed in these cases.

**2) On page 6, in the description of Exome Sequencing results, the sentence "A handful of low-frequency (AF > 0.1%) frequency variants appeared more common in VPSPr cases than controls..." has an extra frequency and should instead read "A handful of low-frequency (AF > 0.1%) variants appeared more common in VPSPr cases than controls..."**

We have deleted the extra "frequency".

**Reviewer #2 (signed Laura Manuelidis)**

**1) BLOTS: The single inadequate caption and prion protein blots are buried in the supplement. These blots**
**belong in the results so fair assumption of a new VPSPr classification can be evaluated. Critical data for each blot**

**(patient age, spongiform changes), PK concentrations with incubation times, other protein controls (as actin) and**
**numbered markers are needed to make these blots comprehensible to non-TSE specialists. Aside from the poor**
**technical quality, the blots do not support their assumption all the cases studied are consonant with infectious sCJD. I assume all these patients have dementia (not stated) and thus should be show obvious PK digested PrP (PrP-**
**res, PrP amyloid) as is known for all clinical cases of sCJD with typical spongiform changes (none shown here).**

The statement "the blots do not support their assumption all the cases studied are consonant with infectious sCJD" shows that the reviewer has not understood the paper. The cases are not sCJD, they are VPSPr, which is a different type of prion disease.

**Six of the 10 supplemental blots are copied below for illustration. Patient #1 does not show the typical sCJD**
**banding pattern (down arrow); compare this VPSPr case to T1, T2 lanes with standard sCJD band patterns. Other**
**blots are also problematic (see below). Why is it necessary to load 10x T1 vs T2 if both are 10% homogenates loaded**
**“ul TH (10%)”? T1 & T2 lanes show a minor difference in the lowest band. Is this due to a PrP mutation?**
**Gel #s 2,3,4 & 5 do not show the top 27kDa band of the sCJD controls. Gel 6 completely lacks any typical**
**PrP-res bands, even at 30ul 10% brain load. Additioaly, cerebellar lanes (C), marked with purple dots in blots 3&5,**
**show more PrP-res than cortical regions. Moreover, in blot #5 (middle C lane), shows the T2 sCJD bands. Cerebellar**
**pathology is not in seen in sCJD but in GSS & growth hormone cases. The normal brain blot #5 (red bar) shows a**
**27kd band of PrP, and marker proteins in #5 are positive for PrP. Red lines in #3 & 6 blots denote lanes with no**
**definitive PrP-res. Sharp PK band at 29kd (red dot) seen in #2 gel (not noted), nor detection method.**

These blots show that, in contrast to sCJD, these VPSPr cases have very little protease-resistant $PrP^{Sc}$. This is consistent with the classification of VPSPr established by Zou et al 2010 and now used routinely for 15 years in prion surveillance.

The blots are not critical to the message of this paper and were added only in response to this reviewer's request on the first round of review. We suggest removing this supplementary material for publication.

**2) FAMILIAL and GENETIC sCJD or NON-INFECTIOUS DEMENTIAS: Given the negative or questionable PrP-res results above for the VPSPr cohort, more information is needed on VPSPr "unique patterns" of spongiform changes and concomitant b-amyloid plaques or tau in these cases (p3). It is likely that questionable PrP-res cases belong in the non-infectious AD spectrum of diseases, especially since only 6 of the total showed infectivity verified by a second serial passage. Rather than acknowledge this limited data, or that more infectivity studies could help define the 67 VPSPr cases, the authors dismiss infectivity studies: "Transmission to humanized mice has been challenging9,10, although the transmissibility of VPSPr has been established in bank voles11". This is misleading and needs the phrase "in only a few of the tested cases (n=6 of 32)", as summarized in my 1st review.**
**The only substantial PrP genetic change included in the paper are MM to VV variants and differences in this**
**set are minor and correspond to standard sCJD (PrP-res positive) cases. This is consonant with most other PrP**
**mutations that affect differences in susceptibility (as to bovine TSE) rather than familial disease. The "familial"**
**argument for VPSPr is weak because it is also based on non-specific neurologic disease in some family members and**
**is only "nominally higher than the 12.1% reported in sporadic CJD [24]" and the data do not support the**
**speculation that VPSPr might be a form of "genetic prion disease" (new p2). If anything, the data indicate**
**susceptibility. Truly genetic (inherited) cases of prion disease are extremely rare, e.g, FFI (aa178) and aa198 Indiana**
**kindred (Brain Pathol. 1995, PMID: 7767492). The addition that "a GWAS has since shown that sCJD has relatively**
**low heritability (h2 = 0.24 to 0.26) [35]" on p12 now gives a better perspective on "familial" disease.**
**Infectious sCJD is consistent pathologically (including PrP-res) in patients and in transmission studies**
**regardless of PrP genotype. sCJD is clearly a very different agent from kuru, BSE and the Asiatic agent-strain of CJD**
**(PNAS. 2009 PMID:19633190; Virulence. 2011, PMID: 21527829), and there is no sCJD maternal transmission in**
**long lived animals (Proc Soc Exp Biol Med. 1979 PMID: 368815). In fact, proven genetic changes in PrP that can**
**cause PrP disease, rather than modify susceptibility, are probably much lower 15% (etimated ~40 years ago).**
**Finally, because the VPSPr classification includes starkly heterogeneous cases, including Alzheimer's**
**Disease (AD) and others, it is essential to include other relevant markers for dementia to sort out a more defined set**

**of cases. For example, major TDP43 changes define “LATE” AD cases exhibiting dementia with rare b-amyloid**
**plaques. TDP43 and other specific markers should be excluded before concluding the 67 cases here are sCJD. Yet the**
**last sentence states “Overall, our study supports the classification of VPSPr as a sporadic prion disease.”**

The sentence "other specific markers should be excluded before concluding the 67 cases here are sCJD" again shows the reviewer's lack of understanding of the manuscript. These 67 cases are <u>not</u> sCJD. They are VPSPr. VPSPr is a category of prion disease established in 2010. Its neuropathological characteristics and transmissibility to animals have been studied elsewhere and are not the topic of this manuscript. This manuscript is about potential genetic risk factors for VPSPr.

Further, the claim that "most other PrP mutations that affect differences in susceptibility (as to bovine TSE) rather than familial disease" is rooted in the reviewer's belief that what we call prion disease is caused by a nucleic acid-based pathogen such as a virus, and therefore, that there cannot be genetic *causes*, only *susceptibility* loci. That's not true. Overwhelming evidence indicates that PRNP mutations *do* cause prion disease, and some are very highly penetrant (Minikel 2016, PMID: 26791950). This is consistent with current genetic counseling guidelines (Goldman & Vallabh 2022 PMID: 35819418).

**SUMMARY: Does the data support the conclusion that VPSPr is a sporadic prion disease?**
**NO**
**Is there convincing evidence for a homogenous VPSPr disease classification with strong familial features?**
**NO**
**Have other forms of late-onset dementia in the AD spectrum been excluded?**
**NO**

This reviewer does not believe in prions and did not understand the manuscript as evidenced by the points above.

**The authors do show convincing negative data for lack of non-coding DNA mutations around the PrP gene locus in this poorly characterized group of 67 patients. While this limited negative data may be useful for Prion (TSE) investigators, it is of very limited genetic and general interest. It is also of concern to this reviewer that patients with AD or other dementia subtypes may be misdiagnosed as variable prion disease (VPSPr) and receive suboptimal advice and treatment.**

We believe that the reviewer's limited enthusiasm for our work is due to her personal conviction that prions do not exist, combined with fundamental misattributions about what this paper is intended to show.
