# Supplementary material for: Search for a genetic cause of variably protease-sensitive prionopathy
Source: PLoS Pathog. 2025 Aug 1;21(8):e1013343. doi: 10.1371/journal.ppat.1013343 (PMC12327619; doi:10.1371/journal.ppat.1013343)
Supplement: Peer review history

# Author comment: Author response to Decision Letter 0

**DOI:** 10.1371/journal.ppat.1013343.r001

**Transfer Alert**

This paper was transferred from another journal. As a result, its full editorial history (including decision letters, peer reviews and author responses) may not be present.

30 Jun 2025

Attachment Submitted filename: R2R_combined_round1_and_round2.pdf

# Editor report: Decision Letter 0

**DOI:** 10.1371/journal.ppat.1013343.r002

**Authors:** Surachai Supattapone

2 Jul 2025

Dear Dr Minikel,

We are pleased to inform you that your manuscript 'Search for a genetic cause of variably protease-sensitive prionopathy' has been provisionally accepted for publication in PLOS Pathogens.

Before your manuscript can be formally accepted you will need to complete some formatting changes, which you will receive in a follow up email. A member of our team will be in touch with a set of requests.

Please note that your manuscript will not be scheduled for publication until you have made the required changes, so a swift response is appreciated.

IMPORTANT: The editorial review process is now complete. PLOS will only permit corrections to spelling, formatting or significant scientific errors from this point onwards. Requests for major changes, or any which affect the scientific understanding of your work, will cause delays to the publication date of your manuscript.

Should you, your institution's press office or the journal office choose to press release your paper, you will automatically be opted out of early publication. We ask that you notify us now if you or your institution is planning to press release the article. All press must be co-ordinated with PLOS.

Thank you again for supporting Open Access publishing; we are looking forward to publishing your work in PLOS Pathogens.

Best regards,

Surachai Supattapone

Section Editor

PLOS Pathogens

Surachai Supattapone

Section Editor

PLOS Pathogens

Sumita Bhaduri-McIntosh

Editor-in-Chief

PLOS Pathogens

orcid.org/0000-0003-2946-9497

Michael Malim

Editor-in-Chief

PLOS Pathogens

orcid.org/0000-0002-7699-2064

***********************************************************

Dear Drs. Vallabh and Minikel,

Thank you for transferring your manuscript to PLoS Pathogens. Based on the reviews you have already received at PLoS Genetics and the revisions you have made based on those reviews, we are happy to accept the manuscript for publication at PLoS Pathogens.

Sincerely,

Surachai Supattapone

Section Editor

Reviewer Comments (if any, and for reference):
